# Variations of the lung microbiome and immune response in mechanically ventilated surgical patients

**DOI:** 10.1371/journal.pone.0205788

**Published:** 2018-10-24

**Authors:** Ryan M. Huebinger, Ashley D. Smith, Yan Zhang, Nancy L. Monson, Sara J. Ireland, Robert C. Barber, John C. Kubasiak, Christian T. Minshall, Joseph P. Minei, Steven E. Wolf, Michael S. Allen

**Affiliations:** 1 Department of Surgery, University of Texas Southwestern Medical Center, Dallas, Texas, United States of America; 2 Department of Microbiology, Immunology, and Genetics, University of North Texas Health Science Center, Fort Worth, Texas, United States of America; 3 Department of Neurology and Neurotherapeutics, University of Texas Southwestern Medical Center, Dallas, Texas, United States of America; 4 Department of Immunology, University of Texas Southwestern Medical Center, Dallas, Texas, United States of America; 5 Department of Pharmacology and Neuroscience, University of North Texas Health Science Center, Fort Worth, Texas, United States of America; Imperial College London, UNITED KINGDOM

## Abstract

Mechanically ventilated surgical patients have a variety of bacterial flora that are often undetectable by traditional culture methods. The source of infection in many of these patients remains unclear. To address this clinical problem, the microbiome profile and host inflammatory response in bronchoalveolar lavage samples from the surgical intensive care unit were examined relative to clinical pathology diagnoses. The hypothesis was tested that clinical diagnosis of respiratory tract flora were similar to culture positive lavage samples in both microbiome and inflammatory profile. Bronchoalveolar lavage samples were collected in the surgical intensive care unit as standard of care for intubated individuals with a clinical pulmonary infection score of >6 or who were expected to be intubated for >48 hours. Cytokine analysis was conducted with the Bioplex Pro Human Th17 cytokine panel. The microbiome of the samples was sequenced for the 16S rRNA region using the Ion Torrent. Microbiome diversity analysis showed the culture-positive samples had the lowest levels of diversity and culture negative with the highest based upon the Shannon-Wiener index (culture positive: 0.77 ± 0.36, respiratory tract flora: 2.06 ± 0.73, culture negative: 3.97 ± 0.65). Culture-negative samples were not dominated by a single bacterial genera. Lavages classified as respiratory tract flora were more similar to the culture-positive in the microbiome profile. A comparison of cytokine expression between groups showed increased levels of cytokines (IFN-g, IL-17F, IL-1B, IL-31, TNF-a) in culture-positive and respiratory tract flora groups. Culture-positive samples exhibited a more robust immune response and reduced diversity of bacterial genera. Lower cytokine levels in culture-negative samples, despite a greater number of bacterial species, suggest a resident nonpathogenic bacterial community may be indicative of a normal pulmonary environment. Respiratory tract flora samples were most similar to the culture-positive samples and may warrant classification as culture-positive when considering clinical treatment.

## Introduction

It is now well understood that the pulmonary tract is inhabited by bacteria under both normal and disease conditions, even though the bacteria may not be detected using traditional culture methods. The recent advent of microbiome profiling has revealed perturbations and dysbiosis of the pulmonary tract [1, 2], especially in regard to disease states such as asthma [3, 4] and chronic obstructive pulmonary disease [5, 6]. Such dysbiosis can ultimately lead to the development of pneumonia in certain populations [7]. Of special interest are mechanically ventilated surgical patients, who can be particularly susceptible to the development of pneumonia. However, a diagnosis of pneumonia in this population is historically problematic, since many of the clinical parameters used for diagnosis are already elevated due to the injuries and other sources of noninfection-related inflammation [8–12]. Most diagnostic criteria for a pneumonia diagnosis include the presence of a bacteria in a culture at a sufficient concentration to suggest infection [13, 14]. However, in many instances, patients will demonstrate clinical signs of pneumonia yet will be culture-negative or have cultures described as “respiratory tract flora.” An analysis of endotracheal aspirates of mechanically ventilated patients demonstrated increased dysbiosis and a reduction of the bacterial microbiome diversity over the time period of intubation. Additionally, the endotracheal microbiome of patients that developed of ventilator acquired pneumonia had increased dysbiosis relative to individuals that did not develop pneumonia when assayed during the first several days of intubation [15]. Molecular methods to quantify the bacterial load in bronchoalveolar lavage fluid [16] and exhaled breath [17] through amplification of the 16S rRNA gene via quantitative real-time PCR have successfully identified patients with microbiologically confirmed ventilator associated pneumonia versus patients with lower bacterial burden. This allowed for a more rapid quantification of bacterial burden relative to the traditional semi-quantitative culture methods [16].

Inflammatory profiles in bronchoalveolar lavage and circulating cytokines have been used to identify [18] as well as discriminate the severity of pneumonia [19]. However, interpreting inflammatory profiles as a diagnostic criterion for pneumonia is difficult in surgical patients, as these patients tend to have an elevated inflammatory state because of their injuries [20] in addition to a potential host response to pneumonia [21].

In this study we sought to examine the microbiome of bronchoalveolar lavage samples from the surgical intensive care unit (ICU) patients, whose immune responses were then compared to those of patients diagnosed with pneumonia using traditional culture methods. The microbiome and inflammatory responses were examined relative to a classification as culture-positive (C+), culture-negative (C-), or respiratory tract flora (RTF). Our hypothesis is that the respiratory tract flora samples were more similar to culture positive lavage samples in their microbiome and inflammatory profile compared to culture negative lavage samples.

## Materials and methods

### Clinical samples

Clinical bronchoalveolar lavage (BAL) samples were collected from mechanically ventilated patients in the surgical ICU at Parkland Memorial Hospital. Inclusion criteria included all adult patients in the SICU who received a BAL and had sufficient lavage fluid to be utilized for research purposes above what was required for clinical diagnosis. Patients were excluded if they were positive for HIV, positive for tuberculosis, under the age of 18, or failed to survive 48 hours past admission. BAL samples were collected using an unprotected BAL catheter in accordance with standard operating procedures developed by the large-scale collaborative project, “Inflammation and the Host Response to Injury” [22]. This BAL procedure is often clinically referred to as a “mini-BAL.” As part of the standard of care, BALs are performed on patients who remain ventilated for over 36 hours (screening) or those with a Clinical Pulmonary Infection Score (CPIS) of ≥6 [23]. Samples for quantitative culture were obtained with a non-bronchoscopic telescoping BAL catheter (BAL-CATH, Kimberly-Clark, Draper UT). After insertion of the catheter five 20cc aliquots of non-bacteriostatic normal saline were instilled and retrieved. The five collections were pooled and a volume of 10–15 ml of the pooled samples were submitted to the pathology laboratory for quantitative culture identification. As part of a de-escalation antibiotic management clinical protocol, administration of antibiotics is stopped if the BAL culture results are negative (< 10^4CFU/ml) or described as RTF. Subsequently, based upon this protocol, C- BAL patients are clinically classified as having SIRS (systemic inflammatory response syndrome) and not pneumonia. Written consent to treat was obtained for the clinical BAL procedure by the practicing physician and documented in the patient’s medical record. Under waived consent excess discarded BAL fluid from the standard of care BAL procedure were utilized for research. Associated clinical data for BAL specimens were additionally collected under waiver of consent. Collection of medical data and utilization of the samples for research with waived consent were approved by the institutional review boards at the University of Texas Southwestern Medical Center (UTSW IRB #062011–135) and University of North Texas Health Science Center.

### BAL sample processing

As described previously [24], upon collection, a portion of the raw BAL fluid was retained for research analysis and stored in a polypropylene tube at 4°C. The remaining BAL fluid was submitted to the Parkland Memorial Hospital Clinical Pathology Laboratory for microbiological identification as part of the standard of care. The raw BAL samples retained for research were picked up twice daily from the surgical ICU and transported to the University of Texas Southwestern Medical Center Surgery Core BSL2+ laboratory, where each sample was given a unique study identification number that was used as part of the de-identification process. A 1 ml aliquot of raw uncentrifuged BAL fluid was placed in a cryovial and stored at -80°C for microbial community analysis. The remaining research fluid was filtered through a 70 micron filter and centrifuged at 1,000 × *g* for 10 minutes to pellet cellular material. Filtered and centrifuged BAL supernatant was placed in a cryovial and stored at -80°C for cytokine analysis.

### Pathology laboratory protocol

The Parkland Memorial Hospital Clinical Pathology Laboratory performed a semi-quantitative culture by using a 1 μl disposable loop to plate raw BAL fluid onto chocolate agar, MacConkey agar, and trypticase soy agar with 5% sheep blood. A gram stain was also prepared by smearing raw BAL fluid onto a glass slide. The BAL samples were pelleted by centrifugation at 3,000 × *g* for 15 minutes and the sediment was processed for acid-fast bacillus culture.

### DNA isolation and 16S rRNA gene library preparation

Raw previously uncentrifuged BAL samples were removed from -80°C storage and allowed to thaw on ice. Then the samples’ cells were pelleted by centrifugation at 15,000 × *g* for 15 minutes. DNA was extracted from the cell pellet using the MasterPure DNA purification kit (Epicentre, Madison, WI) following manufacturer’s instructions with minor modifications. Briefly, cells were lysed using tissue and cell lysis solution with 50 μg/μl proteinase K at 65°C for 15 minutes. RNA was removed using RNase A (5 μg/μl) by incubating at 37°C for 30 minutes. DNA was precipitated using isopropanol and then resuspended in C6 tris-based buffer from the PowerSoil DNA isolation kit (MO BIO [QIAGEN], Germantown, MD). Extracted DNA was stored at -20°C until ready to use. A 16S rRNA gene library was constructed targeting the V4 hypervariable region (515F/806R) [25]. Sample-specific barcodes and Ion Torrent (Thermo Fisher Scientific, Waltham, MA) adapters were synthesized with the forward primer following the IonXpress barcode design (Life Technologies, Carlsbad, CA). PCR was performed using the AccuPrime Taq DNA polymerase high-fidelity system (Life Technologies, Grand Island, NY), consisting of 2.5 μl 10x AccuPrime PCR Buffer II, 1 μl each of 10 μM forward and reverse primer, 1 μl of 50 mM MgSO_4_, 0.1 μl AccuPrime Taq Polymerase High Fidelity, with molecular grade water and template DNA making up a 25 μl reaction mix. The PCR conditions were pre-denaturation at 94°C for 2 minutes, followed by 30 cycles of denaturation at 94°C for 15 seconds, annealing at 52°C for 15 seconds, extension at 68°C for 20 seconds, and a final cycle of 5 minutes extension at 68°C. PCR amplification was performed in triplicate for each sample and visualized on a 1.5% agarose gel with ethidium bromide. Triplicated products were pooled and purified using Agencourt AMpure XP Reagent (Beckman Coulter Genomics, Danvers, MA) following the Ion Torrent protocol. Amplicon size and quantity were assessed using an Agilent DNA 7500 and read on an Agilent Bioanalyzer 2100 (Agilent Technologies, Santa Clara, CA). Purified PCR amplicon were combine together with equimolar concentrations.

### Ion Torrent sequencing

The pooled libraries were diluted to a final concentration of 26 pM in low TE, immobilized on ion sphere particles (ISPs) and amplified using a 400 bp kit on the One Touch II System according to the manufacturer’s protocol. The template positive ISPs were enriched using the Ion Torrent ES. The enriched ISPs were then sequenced using a 316 v2 sequencing chip on the Ion Torrent Personal Genome Machine (Life Technologies, Carlsbad, CA).

### 16S rRNA quantification

The 16S rRNA gene copies in BAL samples were quantified using droplet digital PCR (ddPCR) on a QX200 ddPCR system (Bio-Rad, Hercules, CA). The 22 μl PCR reaction mix contained 11 μl of 2x QX200 ddPCR EvaGreen Supermix, 0.44 μl of (each) 515F and 806R primers (10 μM), 9.02 μl of H_2_O, and 1.1 μl of template DNA. Then 20 μl of PCR reaction solution was mixed with 70 μl of QX200 Droplet Generation Oil for EvaGreen (Bio-Rad) in a DG8 cartridge to generate droplets. About 40 μl of formed droplets were transferred into a 96-well PCR plate, sealed, and amplified in a Bio-Rad C1000 thermocycler under the following conditions: 1 cycle of denaturation at 95°C for 5 minutes, 40 cycles of 95°C for 30 seconds and 52°C for 2 minutes, and 1 final cycle of signal stabilization at 4°C for 5 minutes and 90°C for 5 minutes. Fluorescence signals were read in the QX200 droplet reader and 16S rRNA gene copies were calculated by QuantaSoft software (BioRad). 16s rRNA gene copies were compared between groups with the Kruskal-Wallis test and utilized Dunn’s post-hoc multiple comparison test to determine statistical significance between groups.

### Microbiome data analysis

Microbiome sequence data analysis was performed using a mothur v1.36.1 data-analysis pipeline [26]. Briefly, barcodes and primer sequences were trimmed off and at the same time DNA sequences from individual BAL samples were identified by sample-specific barcodes. Sequences shorter than 100 bp or with a quality score lower than 25 were removed from the dataset. Sequences were aligned against the SILVA reference database. The not aligned sequences as well as redundant sequences were excluded from the dataset using a unique.seqs and precluster command. Chimeras were identified and removed using UCHIME [27]. Operational taxonomic units (OTUs) were defined based on a 97% sequence similarity. Microbial community diversity estimators (Shannon diversity, ACE, and Chao1) were calculated at OTU level. The Greengenes database was used to perform a taxonomic classification of sequences with a minimum 80% confidence level [28]. UniFrac and principal coordinate analysis (PCoA) were conducted to compare the microbial communities among the BAL samples [29]. Analysis of molecular variance (AMOVA) and analysis of similarities (ANOSIM) tests were performed to assess microbial community variances among and within different groups and the similarity among different groups.

### Genetic data availability

The sequences in this study are deposited in the NCBI sequence read archive (SRA) database under the accession numbers SRP082546 and SRP118537. A portion of the C- samples included in this study were processed, sequenced, and reported in a previously published study conducted by Smith *et al*. However, these samples did not have cytokine levels quantified as a part of that study [24].

### Cytokine analysis

Serum cytokines were analyzed with the Bio-Plex Pro Human TH-17 Cytokine panel according to the manufacturer’s recommended protocol with the Bio-Plex MAGPIX Multiplex Reader (Bio-Rad, Hercules, CA). Data were analyzed with Bio-Plex Data Pro software. Samples that were below the limit of detection were included in the analysis with a value of 0.0 pg/ml of the particular cytokine. Cytokine levels were compared between groups with the Kruskal-Wallis test and utilized Dunn’s post-hoc multiple comparison test to determine statistical significance between groups (GraphPad Prism 7). The cytokine profiles of individual samples were analyzed with t-distributed stochastic neighbor embedding (tSNE) [30] implemented in R (version 3.2.3).

## Results

**Fig 1:**
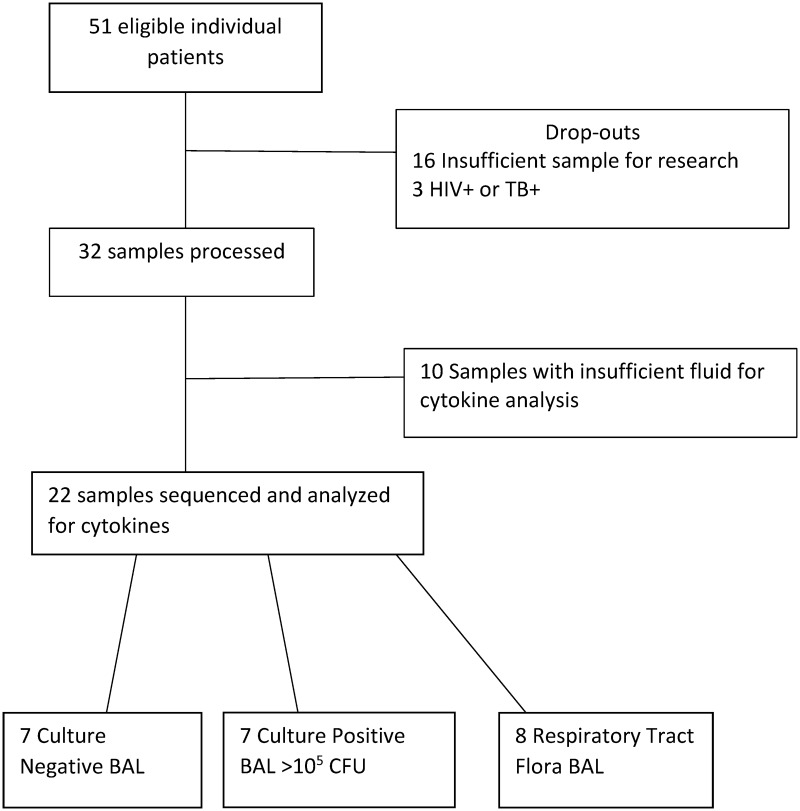
Flowchart of bronchoalveolar lavage samples collected from the surgical intensive care unit.

A total of 22 individual BAL samples were analyzed in this study (Fig 1). These samples were comprised of 7 C+, 7 C-, and 8 RTF samples. Characteristics of each sample group regarding age, gender, prior antibiotic administration, reason for BAL, and days post injury for BAL collection are listed in Table 1. Sequence analysis of 6 of the C- samples were previously reported in Smith et al [24]. Samples were selected sequentially based upon the diagnosis of the pathology laboratory. The majority of samples from antibiotic-treated patients were culture negative (Table 1). A description of the species and bacterial colony forming unit (CFU) abundance for each sample is listed in Table 2. Presence of ventilator acquired pneumonia and/or inflammation using CDC criteria for each patient at the time of BAL is also noted in Table 2. A total of 8 of the 22 samples were from patients who received antibiotic treatment prior to the collection of BAL fluid. Of all the BAL samples, 3 were from patients who received a diagnostic BAL, whereas the remaining 19 samples were collected from patients as part of a routine pneumonia screening protocol in the surgical ICU.

**Table 1:** Demographic data of bronchoalveolar lavage samples stratified by traditional culture results.

Variable	C- (n = 7) Median (IQR); Count (Percentage)	C+ (n = 7) Median (IQR); Count (Percentage)	RTF (n = 8) Median (IQR); Count (Percentage)	P value
Age (years)	40 (26–54)	51 (32–73)	47 (29–57)	0.27
Male gender	6 (86%)	6 (86%)	7 (87%)	0.99
Days Post Injury of BAL	4 (2–11)	2 (2–6)	3 (2–4.5)	0.80
Reason for BAL Collection				
--Diagnostic	2 (28%)	0 (0%)	1 (12.5%)	<0.0001
--Screening	5 (72%)	7 (100%)	7 (87.5%)	
Reason for ICU admission				
Trauma	4	6	5	0.51
Oncology	2	0	2	
CNS	0	1	1	
Other	1	0	0	
Antibiotics at time of BAL	5 (71%)	1 (14%)	1 (12.5%)	0.028
16s Copy Number	24,266(10,860–134,000)*	434,600(134,200–912,000)	343,000(126,866–720,850)	0.006

Quantitative data is presented as median and interquartile range, whereas qualitative data is presented as count and percentage of samples analyzed. Age, gender and days post injury for BAL collection were not significantly different between groups. Kruskal-Wallis test was utilized to compare 16s copy number in GraphPad Prism 7 (p = 0.0057).

Dunn’s post-hoc multiple comparison test was utilized to adjust P-values to determine statistical significance at *P < 0.05.

Culture negative 16s copy numbers were statistically significant between culture positive (adjusted P = 0.0154) and respiratory tract flora samples (adjusted P = 0.0442). 16s copy numbers were not statistically significant between respiratory tract flora and culture positive samples (adjusted P>0.999). Abbreviations: RTF = respiratory tract flora

**Table 2:** Quantification of 16S copies in culture-negative, culture-positive, and respiratory tract flora (RTF) for bronchoalveolar lavage (BAL) samples. Patients who were on antibiotics at the time of BAL are denoted with an asterisk next to BAL ID #. Pathology results are the results of BAL which were used for treatment clinically. Copy number of 16S is denoted at copies per microliter +/- standard error of the mean (SEM).

BAL ID #	Pathology Lab Results	VAP	Inflammation	No Inflammation	16S Copies/μl ± SEM
			No VAP	NoVAP	
186	No growth	No	No	Yes	24,266 ± 624
189*	No growth	No	Yes	No	21,840 ± 706
196	No growth	No	No	Yes	10,860 ± 1,244
197*	No growth	No	Yes	No	6,160 ± 136
201*	No growth	No	No	Yes	252,533 ± 9,534
209*	No growth	No	Yes	No	134,000 ± 5,108
260*	No growth	No	No	Yes	67,533 ± 7,720
184	>100,000 *S*. *aureus*, >100,000 *S*. *pneumoniae*	Yes	No	No	413,000 ± 9,035
195*	>100,000 *Citrobacter koseri*, 40,000 *P*. *aeruginosa*	Yes	No	No	912,000 ± 24,248
204	>100,000 Streptococci (NOT *S*. *pneumoniae* or Enterococcus)	Yes	No	No	78,666 ± 2,130
204B	>100,000 *Pseudomonas*, >100,000 *Enterobacter spp*., 100,000 RTF	Yes	No	No	6,789,333 ± 202,403
206	>100,000 *H*. *influenza*, light RTF	Yes	No	No	669,200 ± 20,047
221	>100,000 *M*. *catarrhalis*, 20,000 *K*. *pneumoniae*	Yes	No	No	434,600 ± 16,985
226	>100,000 *Acinetobacter baumannii/ haemolyticus*, >100,000 RTF	No	Yes	No	134,200 ± 3,113
185	60,000 RTF	No	Yes	No	836,000 ± 42,755
211	50,000 RTF	No	No	Yes	725,800 ± 4,331
224	30,000 RTF	No	Yes	No	209,600 ± 7,480
229	50,000 RTF	No	Yes	No	476,400 ± 2,271
246*	3,000 RTF	No	Yes	No	110,933 ± 7,968
249	2,000 RTF	No	Yes	No	706,000 ± 14,089
254	4,000 RTF	No	Yes	No	174,666 ± 5,484
255	30,000 RTF	No	Yes	No	53,400 ± 6,293

**Fig 2:**
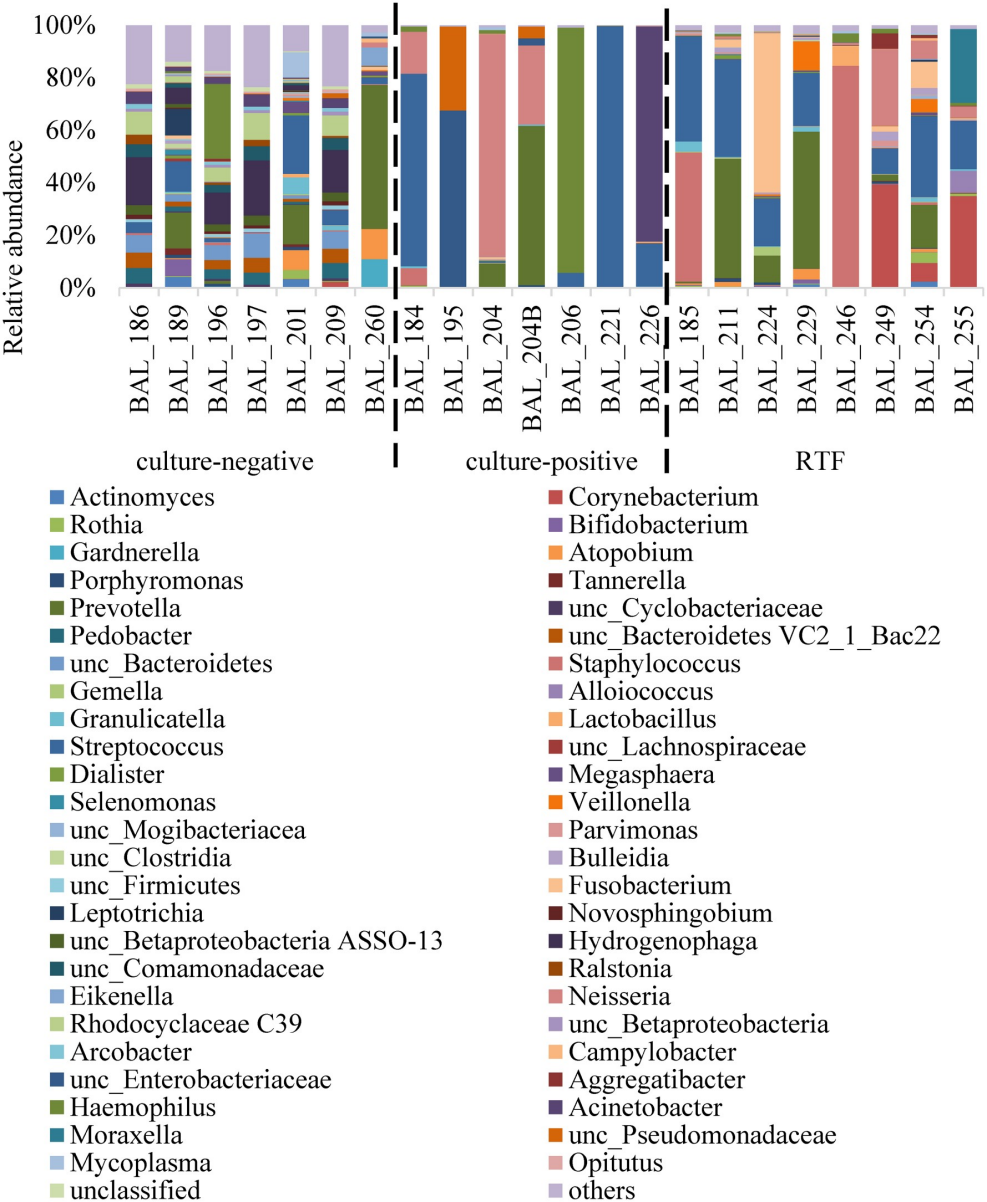
Relative abundance of bacterial genera in culture-negative, culture-positive, and RTF (respiratory tract flora) for bronchoalveolar lavage (BAL) samples. Genus-level taxonomic designations with 2% relative abundance are presented.

A total of 3,864,350 sequences were used for taxonomic classification with a range of 58,963 to 366,076 sequences per sample. Sequences were classified to the lowest taxonomic designation possible, most at the genus level. The relative abundance of bacteria by taxonomic classification level is detailed in Fig 2.

**Fig 3:**
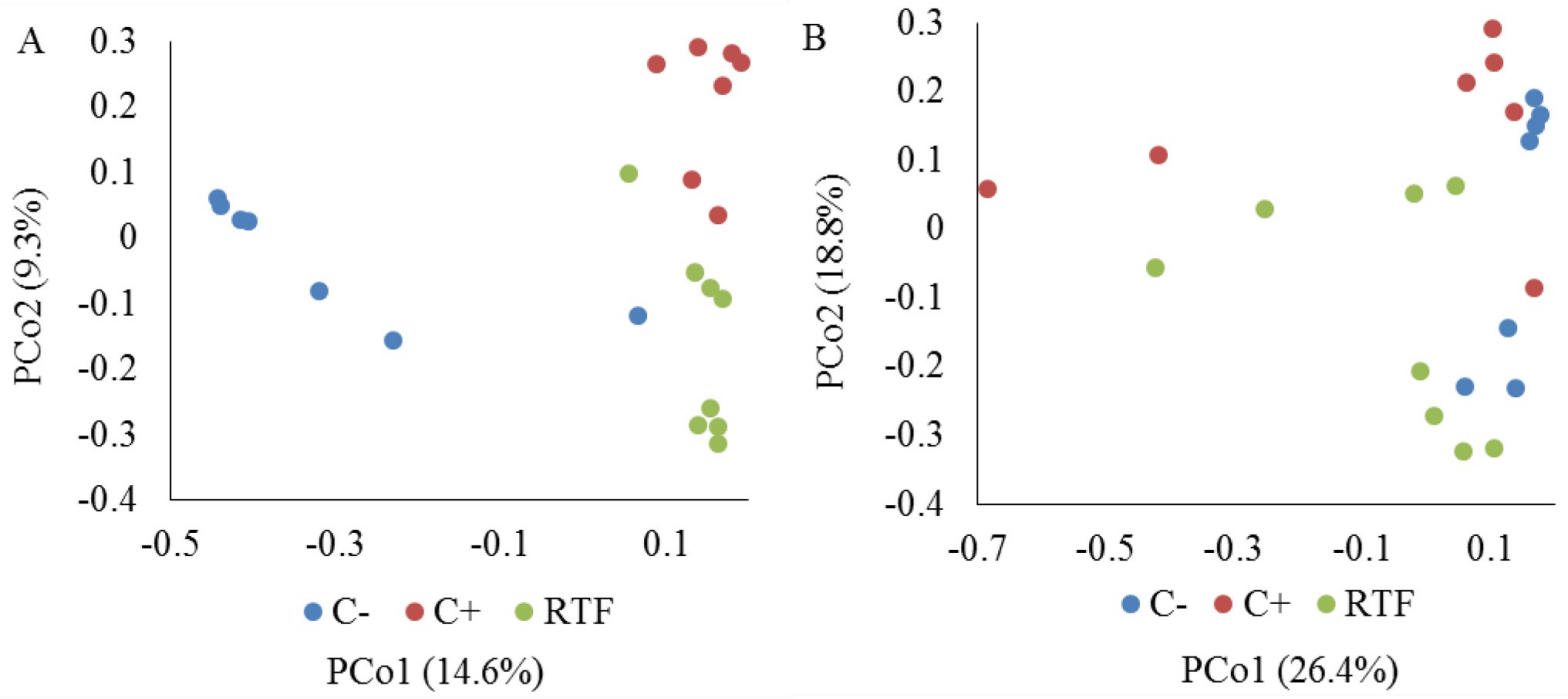
Principal coordinate analysis (PCoA) for bronchoalveolar lavage samples based on unweighted (A) and weighted (B) UniFrac distances. Culture-negative samples (C-) are shaded in blue, culture-positive (C+) in red, and respiratory tract flora (RTF) samples in green.

The diversity of C-, C+, and RTF samples were significantly different from each other (Tukey’s HSD *P* values < 0.01). The C- samples had the highest diversity and C+ samples had the lowest diversity among the three groups (the Shannon-Wiener index for C- was 3.97 ± 0.65, C+ was 0.77 ± 0.36, and RTF was 2.06 ± 0.73 [mean ± standard deviation (SD)]). The C+ samples also had the lowest richness among the three groups (the Chao1 index for C- was 3,696 ± 1,285, C+ was 1,230 ± 383, and RTF was 2,311 ± 1,053 [mean ± SD]; Tukey’s HSD *P* values < 0.05). Both the C+ and RTF samples were dominated by a few species, including several potentially pathogenic microorganisms: *Streptococcus spp*., *Staphylococcus spp*., *Neisseria spp*., and *Fusobacterium spp*. The two BAL samples that exhibited elevated cytokine levels (BAL224 and BAL229) relative to other RTF BAL samples were both comprised of ~20% *Streptococcus*, in addition to ~60% *Fusobacterium* and ~52% *Prevotella*, respectively. A PCoA for the relative abundance data is represented in Fig 3.

The C-, C+, and RTF samples clearly separated from each other on PCoA based on unweighted UniFrac distances, suggesting that the three different groups had very different microbial species (Fig 2A). ANOSIM tests based on unweighted UniFrac distances suggested that microbial communities of C- were significantly different from those of C+ (*r* = 0.84; *P* = 0.0006) and RTF (*r* = 0.74; *P* = 0.0001). The C+ and RTF microbial communities had some overlap (*r* = 0.53; *P* = 0.0003). AMOVA tests based on both unweighted and weighted UniFrac distance showed that microbial population variances between different groups were higher than within groups (*P* < 0.0001 for unweighted, *P* < 0.05 for weighted).

### Total bacterial quantity does not directly correspond to cytokine levels

Each of the BAL samples previously sequenced and measured for cytokine production was also quantified to determine the total number of 16S rRNA gene copies present in the BAL. Droplet digital PCR was used to analyze the C+, C-, and RTF samples. The groups displayed averages of 73,884 (C-), 1,347,286 (C+), and 411,600 (RTF) copies/μl. Table 2 lists the 16S copy number quantification and clinical pathology lab results for each BAL sample. The 16S rRNA copy number of the C- samples was statistically different from both the C+ and RTF groups (p = 0.015 and p = 0.044, respectively). The level of 16S rRNA copy numbers were not significantly different (p>0.999) between the C+ and RTF groups. Only levels of TNF-alpha were significantly correlated with the 16S rRNA gene copy number (*r* = 0.451; *P* = 0.035).

### Cytokines were present at varying levels in the lungs of mechanically ventilated trauma patients

**Fig 4:**
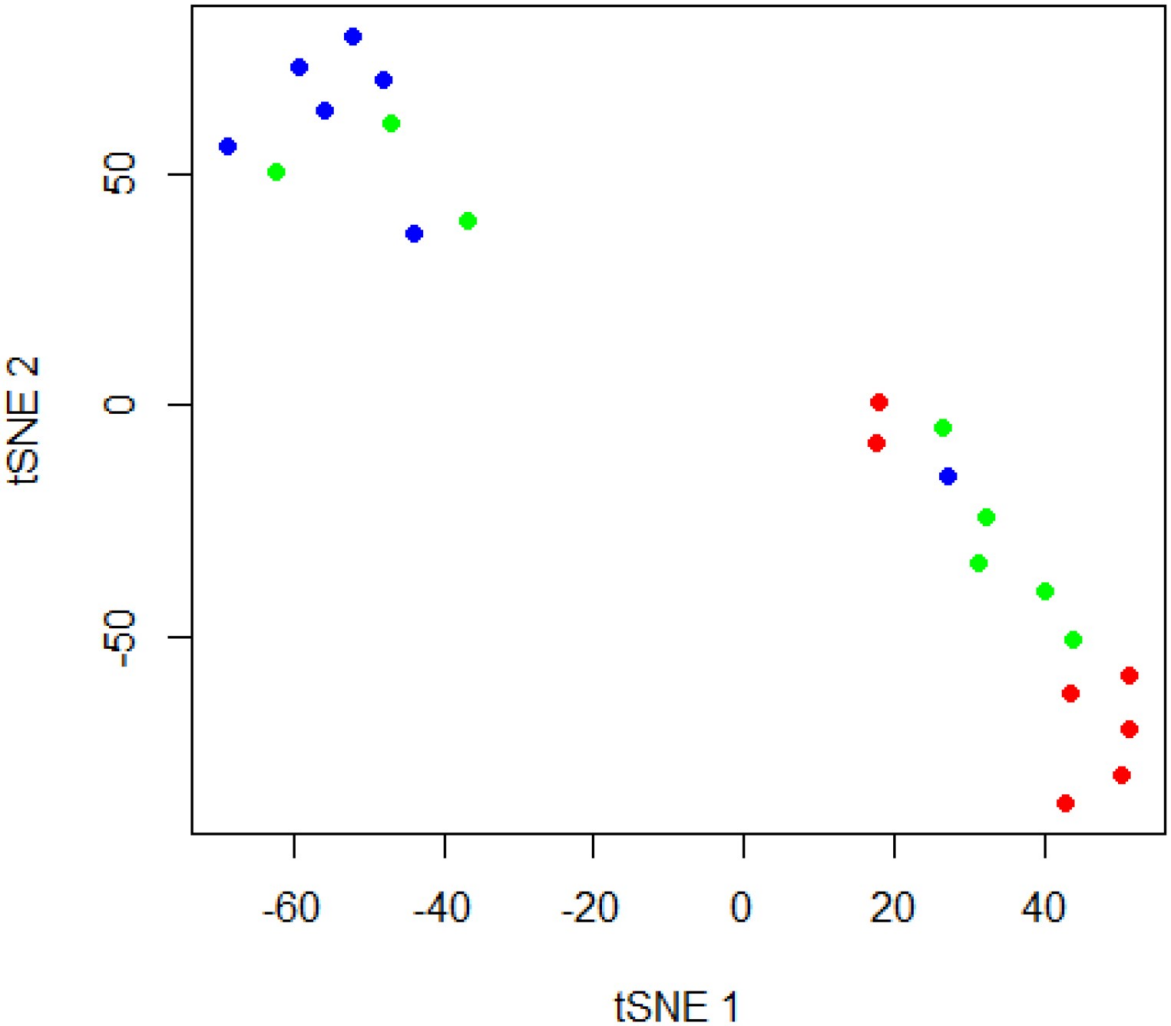
T-distributed stochastic neighbor embedding (tSNE) plot of cytokine profiles from bronchoalveolar lavage samples. Culture-negative (C-) samples are shaded in blue, culture-positive (C+) in red, and respiratory tract flora (RTF) samples in green.

Cytokine levels for all twenty two samples included in this study were measured using the Bio-Plex Pro Human Th17 Cytokine Assay. A majority of cytokine levels in the C- samples were below the limit of detection of the bioplex assay and were included as 0.0 pg/ml as described in the methods. Of the 15 cytokines analyzed, 5 different cytokines (IFN-g, IL-17F, IL-1B, IL-31, TNF-a) expressed significantly different levels between the C+, C-, and RTF groups. The lower limit of detection for these 5 cytokines ranged from 0.27 to 2.98 pg/ml. Cytokine levels of IL-10, IL-17A, IL-21, IL-22, IL-23, IL-25, IL-33, IL-4, IL-6 and sCD40L were detected at very low levels in all of the samples, and were not significantly different between groups (Lower limit of detection range for the non-significant cytokines was between 1.01 to 18.0 pg/ml). Values of the cytokines that were significantly different between groups are shown in Table 3. Cytokine levels in the RTF BAL samples produced cytokine levels more similar to the C+ samples than the C- samples except for IL-1B. Levels of IL-1B were intermediary between C+ and C- samples. Two RTF samples (BALs 224 and 229) continually displayed higher cytokine levels than other samples in the RTF group and several of the C+ samples. A cytokine profile comparison of the 5 significantly different cytokines (IFN-g, IL-17F, IL-1B, IL-31, TNF-a) using t-SNE is represented in Fig 4. Correlations between the number of genera in each sample and cytokine levels were assessed. Genus counts were negatively correlated to levels of IFN-gamma (*r* = -0.502; *P* = 0.0173), IL-17F (*r* = -0.479; *P* = 0.024), IL-1B (*r* = -0.54; *P* = 0.0095), and IL-31 (*r* = -0.527; *P* = 0.0116). Genus counts and levels of TNF-alpha were not significantly correlated (*r* = -0.281; *P* = 0.206).

**Table 3:** Cytokine levels in bronchoalveolar lavage (BAL) of mechanically ventilated trauma patients.

	Culture Positive	RTF	Culture Negative
**IFN-Gamma**	17.45 ± 3.46	15.84 ± 5.03	1.12 ± 0.73*
**IL-17F**	22.03 ± 3.22	21.84 ± 7.96	4.22 ± 2.70*
**IL-1B**	1190.67 ± 340.1	349.69 ± 104.1	46.27 ± 37.13*
**IL-31**	6.11 ± 1.30	5.88 ± 1.74	0.71 ± 0.46*
**TNF-a**	28.49 ± 9.49	42.104 ±22.2	0.8 ± 0.0.32*

Supernatants from raw BALs were measured by multiplex cytokine assay. Samples were divided into three groups based on the results from the clinical pathology lab: culture-negative, culture-positive, and respiratory tract flora (RTF). All samples were run in triplicate. Values are given as pg/ml ± standard error of the mean. Culture-negative and RTF samples were compared against culture-positive samples using Kruskal-Wallis test (overall P = 0.0006).

Dunn’s post-hoc multiple comparison test was utilized to adjust P-values to determine statistical significance at *P < 0.05.

The cytokine levels between culture positive and culture negative samples were statistically different (adjusted P = 0.0016). Cytokine levels between respiratory tract flora and culture positive (adjusted P = 0.1957) or culture negative (P = 0.2526) samples.

## Discussion

Historically, a pneumonia diagnosis in the surgical ICU has been problematic as most patients undergoing mechanical ventilation have clinical symptoms that conflict with most of the diagnostic criteria [31, 32]. Existing clinical tools like the Clinical Pulmonary Infection Score have demonstrated insufficient sensitivity and specificity for pneumonia diagnosis [33]. This can facilitate delays in the diagnosis of ventilator-associated events, such as pneumonia. Additionally, patients who meet clinical criteria for a ventilator-associated event or potentially ventilator-associated pneumonia sometimes remain C- or do not culture a specific pathogen (i.e., RTF). This can become problematic as clinicians are unable to target pathogens that may be the source of a ventilator-associated event or pneumonia.

Previous research demonstrates that the pulmonary tract is not in fact sterile, but is occupied by a wide array of bacterial species even when bacterial growth is absent through traditional culture methods. As expected, bacteria were sequenced from all of the samples in the current study, including samples that did not have viable bacteria through traditional culture techniques.

The C- samples showed the highest level of diversity, which could represent the healthy bacterial relationships within the lung. The majority of C- samples that were sequenced in this study were not dominated by a single bacterial genus. Only one BAL (BAL260) had a genus with a relative abundance above 30% of the sample. In the unweighted PCoA plot, BAL260 clustered with the C+ and RTF samples. In contrast, the C+ samples were all dominated by a single bacterial genus, as might be expected given their C+ status. All C+ samples had a single genus with a relative abundance above 60% of the sample, with a concomitant decrease in overall bacterial diversity. The profile of RTF samples similarly showed decreased diversity compared to C- samples. The relative abundance of bacteria in the RTF samples was often dominated by two to three bacterial genera. These genera are often considered clinically significant and commensal oral flora has been associated with the development of VAP, especially in immunocompromised individuals [34]. However, given the mix of genera that comprise the RTF, and their lower levels of CFUs, patients with RTF are often not treated with antibiotics to resolve the bacterial growth. An additional caveat to relying on the relative abundance in a sample is that one cannot determine if the abundance of a taxa is caused by overgrowth of the dominant genera. Although precautions are undertaken during sequencing to determine adequate sampling based upon number of sequences generated per sample, a taxa with low frequency could potentially appear at less than the 1% abundance threshold if the dominant genera is in a stage of overgrowth.

Mechanical ventilation has been reported to increase dysbiosis of the pulmonary tract. In a study of endotracheal aspirates over the course of ventilation demonstrated that bacterial diversity of aspirates at the familial taxonomic level decreased over the period of mechanical ventilation. Additionally, patients who developed VAP had a larger decrease of bacterial diversity relative to those who did not progress to VAP while under mechanical support. There was not a statistical difference in the change of microbial composition over intubation between patients who developed VAP and patients with colonized airways [15]. Although this appears to counter the diversity differences noted in this paper, Zakharkina et al. presented changes in the diversity of a patient over a period of mechanical ventilation as opposed to the temporal diversity sampling presented herein. Additionally, Zakharkina analyzed the diversity of samples at the familial taxonomic level, whereas the diversity of samples from this cohort are described at the genus level. As one ascends to a higher level of the taxonomic hierarchy, the diversity identified at lower levels (i.e. genus) is condensed into a broader taxonomic category (i.e. family, or phyla). Comparing samples at a higher taxonomic level may underestimate the differences between samples relative to comparing diversity at the genus or species taxonomic level. In the graphical presentation of the relative abundance data, Zakharkina et al subsumed the data into 8 groups. These groups comprised 7 bacterial families and the 8^th^ group was a collection of all other bacterial families. In an examination of the data presented here the mean number of families was 19 in the culture positive, 30 for the RTF group, and 99 in the culture negative group. Reducing the majority of taxa into the “other” grouping may further explain the differences in the diversity observed between Zakharkina et al and the data presented here. Additionally, Zakharkina and colleagues examined the microbiome of tracheal aspirates, and previous publications have suggested that there are varying levels of concordance between endotracheal aspirates and bronchoalveolar lavage fluid for diagnosing VAP [35–38].

Inflammatory cytokines have previously been associated with pulmonary infections. It has been proposed to utilize different cytokines and cytokine profiles to aid in the diagnosis of pneumonia [18, 39, 40]. Among the BAL samples we analyzed, the C+ group exhibited the highest levels of proinflammatory cytokines. This is not surprising as the C+ samples all had significant levels of bacteria detected by traditional culture methods (>10^5^ CFUs). Additionally, the RTF group exhibited elevated cytokines similar to what was seen in the C+ group. The C- group exhibited very low levels of cytokines across all of the cytokines examined in this study. Additionally the RTF and C+ groups were more similar to each other in regards to microbiome profile and cytokine production than they were to the C- group. Using t-SNE to compare cytokine profiles of each sample, the majority of C+ and RTF samples were grouped together. One C- sample, BAL260, grouped with the C+ and RTF samples. BAL260 also was noted to have a microbiome profile that was similar to the C+ and RTF groups. The t-SNE analysis calculates a pair-wise probability matrix based upon the level of similarity between individual samples. These differences in the inflammatory profiles between the C+ and RTF group and the C- group could be attributable to the overgrowth of potentially pathogenic organisms that facilitate an increased immune response. These low levels of cytokines could potentially be used to stratify patients who do not require antibiotic treatment (low cytokine, C-) versus patients in which pneumonia could not be excluded (C+ and RTF with high cytokine levels).

**Fig 5:**
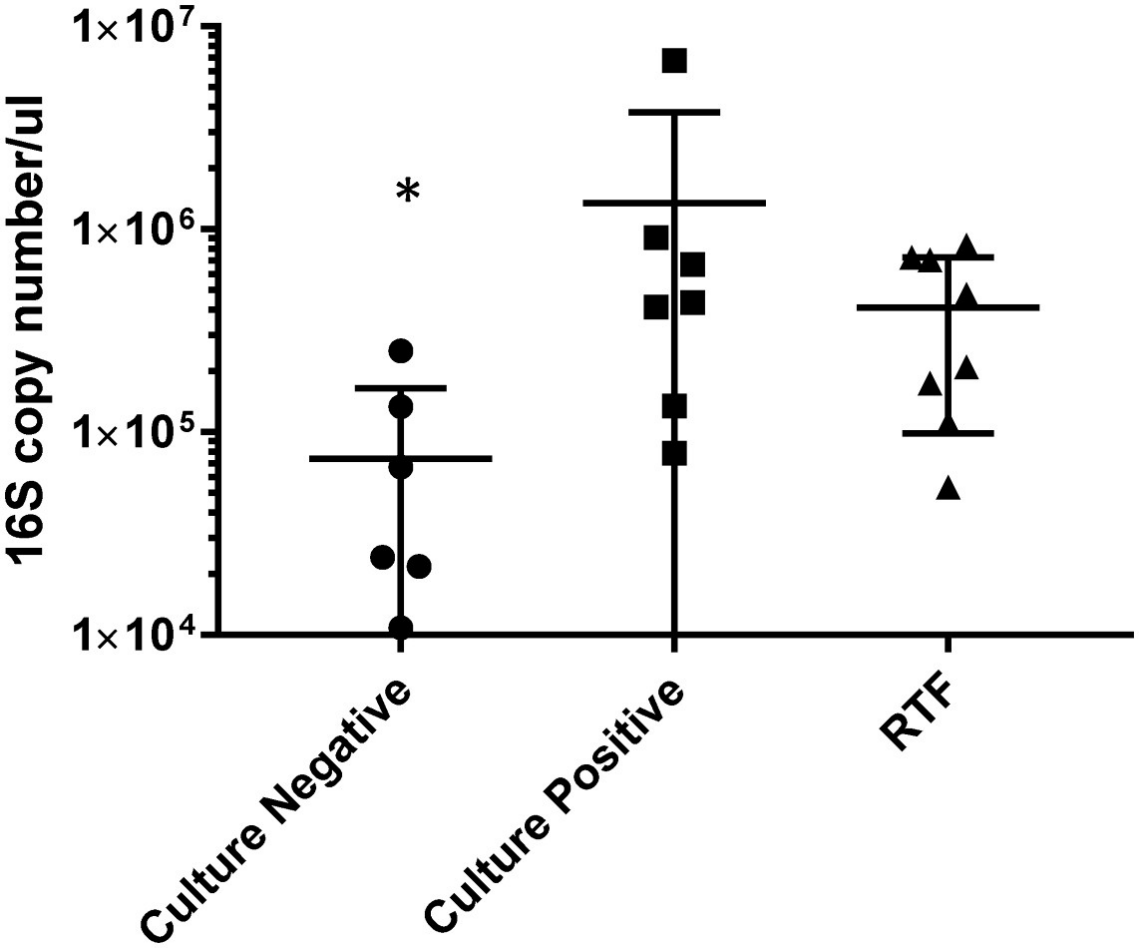
Statistical analysis of 16S rRNA gene copy numbers in culture-negative, culture-positive, and respiratory tract flora (RTF) for bronchoalveolar lavage (BAL) samples. In all, 16S rRNA gene copy numbers from culture-negative, culture-positive, and RTF BAL samples were quantified using droplet digital PCR. Includes values from Table 2 that were compared using a Kruskal-Wallis test in GraphPad Prism 7 (p = 0.0057). Dunn’s post-hoc multiple comparison test was utilized to adjust P-values to determine statistical significance at *P < 0.05. Culture negative 16s copy numbers were statistically significant between culture positive (adjusted P = 0.0154) and respiratory tract flora samples (adjusted P = 0.0442). 16s copy numbers were not statistically significant between respiratory tract flora and culture positive samples (adjusted P>0.999). Error bars are represented as median and inter-quartile range.

Surprisingly, BAL 204B contained over 6 million 16S copies/μl, a measurement 6× greater than BAL 195, which had the next highest number with ~912,000 copies/μl. Although BAL 204B exhibited an extremely high number of 16S copies, its level of cytokine production correspondingly was not the highest of the BALs analyzed. BALs 224 and 229 that displayed high cytokine levels did not contain unusually high 16S copy numbers. While the average copy number in C- samples was significantly lower than the averages of the other two groups, some of the C- samples had numbers equivalent to samples in the other groups. Additionally, there was no significant difference between 16S copy numbers in the C+ and RTF groups (Fig 5). Importantly, these C- samples with higher numbers of 16S copies did not produce equivalent cytokine levels to C+ or RTF samples. Given that the majority of cytokines were not correlated with 16S copy numbers, there are likely other factors driving the immune response besides bacterial abundance. Conway Morris et al utilized a 16S rRNA quantitative real time PCR assay for early detection of patients with microbiologically confirmed VAP. Although the assays utilized are different from the methods utilized here, they found that microbiologically confirmed VAP had a higher bacterial burden compared to culture negative samples [16]. These findings are in line with the data presented here where the CP and RTF groups had significantly higher 16S rRNA levels compared to culture negative samples.

## Conclusions

Overall, the BAL samples that were dominated by a few genera (C+ and RTF) were more likely to produce high levels of proinflammatory cytokines. These data suggest that the relative abundance of bacteria present in the lungs (as determined by BAL) may be linked to cytokine production. Total microbial diversity was also similar between C+ and RTF samples, both of which were lower than C- samples. Taken together, culture samples reported as RTF had more in common with C+ samples than with C-, suggesting that they may represent a higher risk for pneumonia (as currently defined clinically) in this patient cohort. Along these lines previous examinations of commensal oral flora (by traditional culture methods) have been associated with the development of VAP in the ICU [34]. This may warrant considering whether patients with bacteria designated as RTF should be clinically treated similar to patients who are C+.

This study suffers from several limitations. Although the mini-BAL is commonly used for clinical diagnosis of pneumonia it is considered a blind lavage. A limitation of utilizing this style of lavage catheter is that it is not possible to know where the catheter is lodged, making it difficult to ensure that the samples come from the distal airway and is not contaminated with samples from the more proximal airway. Additional limitations of this study include that it is a single center observational study, encompasses a relatively small number of samples, and may not have broad applicability when applied to a larger sample of ventilated patients.

## Abbreviations

AMOVA: Analysis of molecular variance
ANOSIM: Analysis of similarities
BAL: Bronchoalveolar lavage
C-: Culture-negative
C+: Culture-positive
CFU: Colony forming unit
CPIS: Clinical Pulmonary Infection Score
ddPCR: Droplet digital PCR
ICU: Intensive care unit
ISPs: Ion sphere particles
OTUs: Operational taxonomic units
PCoA: Principal coordinate analysis
RTF: Respiratory tract flora
SD: Standard deviation
SIRS: Systemic inflammatory response syndrome
SRA: Sequence read archive
tSNE: t-distributed stochastic neighbor embedding
